# Changes in swallowing response on patients undergoing chemoradiotherapy for head and neck cancer

**DOI:** 10.1007/s00520-024-09134-6

**Published:** 2025-01-14

**Authors:** Nao Hashida, Motoyuki Suzuki, Kiyohito Hosokawa, Yukinori Takenaka, Takahito Fukusumi, Norihiko Takemoto, Hidenori Tanaka, Koji Kitamura, Hirotaka Eguchi, Masanori Umatani, Itsuki Kitayama, Masayuki Nozawa, Chieri Kato, Eri Okajima, Hidenori Inohara

**Affiliations:** 1 Swallowing Center, Osaka University Hospital, 2-15, Yamadaoka, Suita City, Osaka 565-0871 Japan; 2 Department of Otorhinolaryngology-Head and Neck Surgery, Osaka University Graduate School of Medicine, 2-2, Yamadaoka, Suita City, Osaka 565-0871 Japan

**Keywords:** Acute side effect, Swallowing response, Swallowing response, Delayed swallowing initiation

## Abstract

**Purpose:**

Chemoradiotherapy (CRT) for head and neck cancer (HNC) often causes dysphagia. The risk of dysphagia increases during CRT tends to become more severe after finishing CRT, and persists for a few weeks thereafter. Thus, understanding the changes in swallowing physiology during and immediately after CRT is essential. This study aimed to clarify the changes in the swallowing response during and early after CRT and identify associated factors.

**Methods:**

This retrospective study enrolled 107 patients with HNC who underwent CRT. We measured pharyngeal delay time (PDT) and laryngeal elevation delay time (LEDT) as indicators of the timing of the swallowing response at three time points: at CRT initiation (baseline), at 40-Gy irradiation during CRT (mid-CRT) and within 2 weeks following the completion of CRT (early post-CRT) as primary outcomes; and subgroup analyses based on clinical parameters, such as tumor sites, T stage, N stage, and opioid use at 40-Gy irradiation as secondary outcomes.

**Results:**

Both PDT and LEDT were significantly prolonged between baseline and mid-CRT (PDT: *p* = 0.003, LEDT: *p* = 0.002) and between baseline and early post-CRT (PDT, *p* = 0.001; LEDT, *p* < 0.001). N2c/N3 and opioid use at 40-Gy irradiation showed prolonged PDT and LEDT at mid-CRT and early post-CRT.

**Conclusion:**

PDT and LEDT were prolonged at mid-CRT irradiation and further extended at early post-CRT. Additionally, N2c/N3 involvement, which typically necessitates bilateral neck irradiation fields and opioid use at mid-CRT, may constitute as risk factors for a delayed swallowing response.

## Introduction

Dysphagia is a common complication after head and neck cancer (HNC) treatment, and may lead to dehydration, malnutrition, lower quality of life, aspiration pneumonia, choking, and/or premature death [1].

Chemoradiotherapy (CRT), one of the standard treatment modalities for HNC, has been associated with enhanced local control in the affected region and improved overall survival, along with the added benefit of organ preservation [2]. Despite these substantial advances, dysphagia remains a major concern as both acute and late complications of CRT [3]. The severity of dysphagia can vary, with mucositis, pain, copious mucous production, xerostomia, and soft tissue edema occurring within 4–5 weeks after CRT initiation. These conditions may lead to aspiration, malnutrition, and feeding tube dependency [4]. Moreover, these effects become much more pronounced after completing 7 weeks of CRT [4].

Previous studies have shown that CRT causes laryngeal sensory disturbance, deteriorates the delayed swallowing timing or swallowing response, and limits laryngeal elevation and pharyngeal wall constriction, which contribute to dysphagia [5, 6]. Eisbruch et al. reported delayed swallowing response in 36% of patients with HNC before treatment, 30% of patients 1–3 months after treatment, and 62% of patients 6–12 months after CRT [7]. Moreover, pharyngeal delay time (PDT) was prolonged in patients with an average of 153 days after RT compared with those with an average of 8 days after RT initiation [8]. By contrast, several studies showed that the swallowing response was unchanged 1–3 months after treatment compared with that before treatment [7, 9], and Ohkoshi reported that delayed swallowing response improved 3 months after treatment compared with that before treatment [10]. Acute side effects often occur during CRT, and the risk of dysphagia increases simultaneously. In addition, the side effects and dysphagia are more severe after finishing CRT and persist a few weeks thereafter. Thus, understanding the changes in swallowing physiology during and immediately after CRT, particularly within 2 weeks, is essential; however, no studies have addressed the changes in swallowing response during and early post-CRT or identified the risk factors for dysfunctional swallowing responses.

Thus, this study clarified the changes in the swallowing response at 40-Gy irradiation during CRT, a period when acute side effects such as mucositis, pain, and dysphagia often begin to occur, and early after CRT, a period when patients commonly experience severe swallowing related side effects [4] and identified the factors associated with these changes.

## Materials and methods

### Patients

This retrospective, observational study included patients with HNC who underwent curative CRT at a university-affiliated hospital between January 2019 and August 2023. We excluded patients with a history of HNC treatment, those who underwent pre-CRT surgery or chemotherapy, and those who opted out of participation. Pre-CRT biopsy was not an exclusion criterion. Patients were not routinely provided with speech and swallowing therapy. If otorhinolaryngologists and speech-language pathologists determined that patients had dysphagia and required swallowing rehabilitation, they were offered speech and swallowing therapy based on the results of the VFSS and the clinical condition of each patient.

### Assessment of swallowing response

We conducted a videofluoroscopic swallowing study (VFSS) to evaluate swallowing function at three time points: at CRT initiation (baseline), at 40-Gy irradiation during CRT (mid-CRT), and within 2 weeks following the completion of CRT (early post-CRT). As part of the standard protocol, swallowing function was routinely assessed using VFSS for all patients undergoing CRT. Our protocol included two trials of lateral views of swallowing with 5-ml thin contrast medium, with the better of the two swallowing trials selected for analysis. As a primary outcome, pharyngeal delay time (PDT) and laryngeal elevation delay time (LEDT) were measured by a single speech-language pathologist with more than 10 years experiences [11, 12]. VFSS was recorded at 7.5 frames/s; thus, one frame was approximately 0.13 s. PDT and LEDT were quantified in terms of frame numbers. PDT and LEDT were related to the timing of the swallowing process and associated with the onset of the pharyngeal phase of swallowing. PDT was measured from the time the head of the bolus passes the ramus of the mandible until the onset of laryngeal elevation. LEDT was measured by calculating the time period from when a bolus head reaches the bottom of either side of the pyriform sinus until the larynx is elevated to the highest position.

To evaluate the reliability of the measurements, inter-rater reliability was assessed using the PDT and LEDT data from 32 randomly selected VFSS by another speech-language pathologist who was blinded to the characteristics of the patients. We assessed inter-rater reliabilities using the intraclass correlation coefficient (ICC) (2, 1).

### Other parameters

We evaluated swallowing safety with the penetration aspiration scale (PAS), an eight-point scale that evaluates airway invasion based on the VFSS [13]. Patients were divided into three groups: a PAS score of 1 indicates that no material enters the airway; scores 2–5 indicate material penetration; and scores ≥ 6 indicate tracheal aspiration of material. We also recorded the functional oral intake scale (FOIS) score at starting of CRT, ranging from level 1 (nothing by mouth) to 7 (total oral diet with no restrictions) [14].

### Statistical analysis

Repeated measure analysis of variance (ANOVA) was employed to evaluate the changes in PDT and LEDT. Furthermore, post hoc group comparisons were performed in cases of significant differences. Group comparisons were adjusted using Holm’s method. To clarify variations in the changes in PDT and LEDT, subgroup analyses were conducted based on clinical parameters, such as tumor sites, T stage (T4 or T0–T3), N stage (N0–N2b or N2c–N3), and opioid use (use or nonuse) at mid-CRT. Multivariate linear regression was performed for changes in PDT and LEDT from baseline to early post-CRT with age, sex, T stage (T4 or others), N stage (N0–N2b or N2c–N3), tumor sites (oropharynx or others), opioid dosage, and absence of tube feeding experience during the CRT. The dichotomization of T stage and N stage into separate groups was based on the irradiation field required for treatment. Advanced T stage tumors require more extensive irradiation of the primary tumor site, while advanced N stage cases necessitate bilateral neck irradiation. Opioid dosage was documented in terms of the daily oral morphine equivalent (OME) dose using standard dose conversion calculations [15]. Moreover, age- and sex-adjusted ordinal logistic regression for PAS (score 1: no penetration or aspiration, score 2–5: penetration, or score > 6: aspiration) was conducted to clarify the influence of PDT and LEDT on swallowing function at each time point. Statistical significance was set at *p* value less than 0.05.

### Ethical declarations

This study followed the ethical standards in the 1964 Declaration of Helsinki and its later amendments and was approved by the ethics committee of Osaka University Hospital (approval number 16329–2). The ethics committee of our hospital waived the need for informed consent because of the retrospective nature of the study. However, comprehensive informed consent was obtained from all patients prior to treatment. Additional opt-out technique through an online announcement on the hospital’s webpage was used to provide an opportunity for patients to withdraw in the study.

## Results

### Patients’ characteristics

A total of 112 patients met the inclusion criteria. Of the 112 patients, five were excluded because of missing data, resulting in 107 patients subjected for analysis. Table 1 presents the patient’s characteristics. The mean age ± standard deviation (SD) was 66.3 ± 8.7 years, and 16 (15%) were women. The most common tumor sites were the hypopharynx (38%), oropharynx (32%), and larynx (14%). Moreover, 99 patients could eat orally (FOIS score > 4), whereas eight patients required nasogastric or gastrostomy tube feeding without any oral intake at CRT initiation. Early post-CRT VFSS was conducted on a median of 8 days (interquartile range, 5.75–11) following the last irradiation.

**Table 1:** Patient characteristics

		*N* = 107
Age, mean ± SD		66.3 ± 8.7
Sex, *n* (%)	Female	16 (15.0)
Tumor site, *n* (%)	Hypopharynx	41 (38.3)
	Oropharynx	34 (31.8)
	Larynx	15 (14.0)
	Nasopharynx	7 (6.5)
	Nasal cavity/sinuses	6 (5.6)
	Unknown	4 (3.7)
T stage, *n* (%)	T1–T3	76 (71.0)
	T4	31 (29.0)
N stage, *n* (%)	N0–N2b	75 (70.1)
	N2c or N3	32 (29.9)
FOIS score at admission (%)	1	8 (7.5)
	5	1 (0.9)
	6	15 (14.0)
	7	83 (77.7)
Opioid use at mid-CRT, *n* (%)		36 (33.6)

*SD*, Standard deviation; *FOIS*, functional oral intake scale; *PAS*, penetration aspiration scale

### Swallowing assessment and swallowing responses

During the VFSS at baseline, nine patients had aspiration (PAS score > 6), and 13 had penetration (PAS score, 2–5). At mid-CRT, 11 patients had aspiration, and 16 had penetration. In the early post-CRT phase, 25 patients had aspiration, and 22 had penetration. At the mid-CRT, 26 patients required nasogastric or gastrostomy tube feeding, and this number increased to 55 in the early post-CRT. For inter-rater reliability, the measurements of PDT and LEDT had an ICC (2, 1) of 0.918 (95% CI: 0.842 to 0.958) and 0.823 (95% CI: 0.61 to 0.917), respectively.

**Fig. 1:**
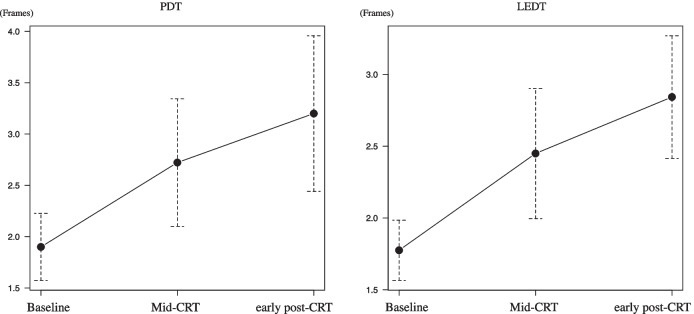
Changes in PDT and LEDT at baseline, mid-CRT, and early after CRT. Mean PDT and LEDT were 1.9 and 1.79 at baseline, 2.7 and 2.45 at mid-CRT, and 3.2 and 2.84 at early after CRT

In conjunction with the repeated-measures ANOVA results, Fig. 1 shows the changes in PDT and LEDT at three measurement points (baseline, mid-CRT, and early post-CRT). Significant differences were found between baseline and mid-CRT (PDT, *p* = 0.003; LEDT, *p* = 0.002), and between baseline and early post-CRT (PDT, *p* = 0.001; LEDT, *p* < 0.001). Although between mid-CRT and early post-CRT exhibited similar trends, PDT was not significantly different (*p* = 0.34). Conversely, LEDT demonstrated a significant difference (*p* = 0.04).

**Fig. 2:**
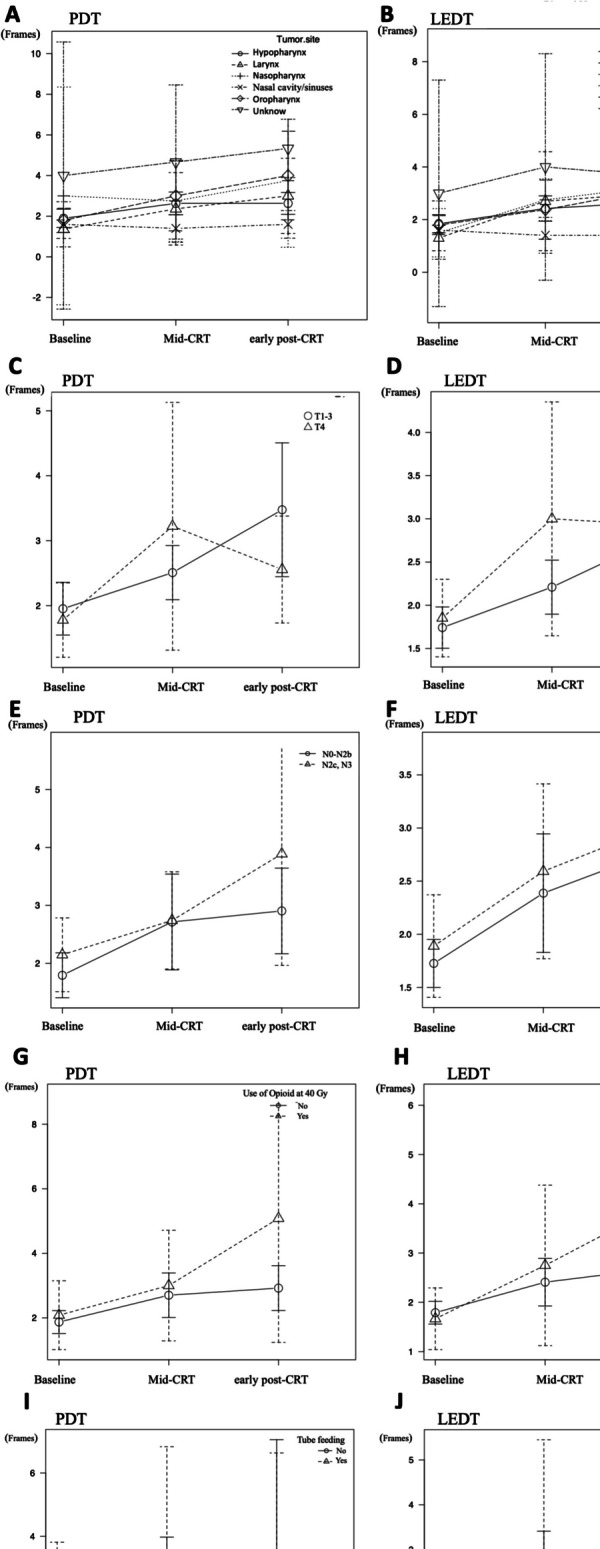
Changes in PDT and LEDT of subgroup analysis at baseline, mid-CRT, and early post-CRT. Based on tumor sites (**A**, **B**), T stage (**C**, **D**), N stage (**E**, **F**), opioid use at 40 Gy (**G**, **H**), and tube feeding experience (**I**, **J**)

Figure 2 depicts the changes in PDT and LEDT in the subgroup analysis, indicating that nasal cavity/paranasal sinus tumors extend neither in PDT nor in LEDT. Regarding the T stage, T4 had no significant association with prolonged PDT and LEDT. N2c/N3 showed a tendency for increased PDT from mid-CRT to early post-CRT; however, this trend was not observed in LEDT, and N1–N2b and N2c/N3 showed similar patterns. The opioid use group showed a noticeable prolongation in both PDT and LEDT from mid-CRT to early post-CRT. Additionally, the absence of tube feeding experience during CRT was not significantly associated with prolonged PDT and LEDT.

In multivariate linear regression, opioid dosage at mid-CRT was a significant factor for changes in both PDT (*p* = 0.023) and LEDT (*p* = 0.001) (Table 2). Table 3 shows that both age- and sex-adjusted PDT (beta =  − 0.39, *p* < 0.001 and beta =  − 0.16, *p* = 0.03, respectively) and LEDT(beta =  − 0.44, *p* < 0.001 and beta =  − 0.20, *p* = 0.03, respectively) were significant factors associated with swallowing safety with PAS score at mid-CRT and early postCRT point, although both PDT and LEDT at the baseline were not significant factors in ordered logistic regression.

**Table 2:** Multivariate linear regression for PDT and LEDT changes from baseline to early post-CRT

	Beta	95% CI	Std. error	*T* value	*p* value
*For PDT change*					
Intercept	− 3.00	− 9.97–3.06	3.05	− 0.98	0.33
Age	0.04	− 0.04–0.13	0.04	0.98	0.33
Sex	1.01	− 1.12–3.14	1.07	0.94	0.35
T4	− 1.11	− 2.70–0.48	0.8	− 1.39	0.17
N2b–N3	− 0.23	− 1.91–1.45	0.84	− 0.27	0.79
Oropharynx tumor	1.24	− 0.29–2.77	0.77	1.61	0.11
Dosage of opioid at mid-CRT	0.04	0.01–0.08	0.02	2.31	0.023
*For LEDT change*					
Intercept	− 0.11	− 3.45–3.24	1.68	− 0.06	0.95
Age	0.01	− 0.04–0.06	0.02	0.35	0.73
Sex	0.34	− 0.84–1.51	0.59	0.57	0.57
T4	− 0.11	− 0.99–0.77	0.44	− 0.25	0.80
N2b–N3	− 0.6	− 1.53–0.32	0.47	− 1.3	0.20
Oropharynx tumor	0.32	− 0.52–1.16	0.42	0.76	0.45
Opioid dosage at mid-CRT	0.03	0.01–0.05	0.01	3.39	0.001

*CRT*, Chemoradiotherapy; *PDT*, pharyngeal delay time; *LEDT*, laryngeal elevation delay time; *CI*, confidence interval

The opioid dose was recorded with daily oral morphine equivalent (OME) dose using standard dose conversion calculations

**Table 3:** Age- and sex-adjusted ordered logistic regression for swallowing safety based on PAS scale

	Beta	95% CI	Std. error	*p* value
*For PAS at the baseline*				
Model 1				
PDT at CRT initiation	− 0.28	− 0.59–0.02	0.14	0.051
Model 2				
LEDT at CRT initiation	− 0.29	− 0.78–0.21	0.23	0.21
*For PAS at mid-CRT*				
Model 1				
PDT at40-Gy irradiation	− 0.39	− 0.64– − 0.18	0.11	< 0.001
Model 2				
LEDT at 40-Gy irradiation	− 0.44	− 0.71– − 0.20	0.13	< 0.001
*For PAS at early post-CRT*				
Model 1				
PDT early after CRT	− 0.16	− 0.32– − 0.03	0.07	0.03
Model 2				
LEDT early after CRT	− 0.20	− 0.41– − 0.01	0.09	0.03

*PAS*, Penetration aspiration scale; *CRT*, chemoradiotherapy; *PDT*, pharyngeal delay time; *LEDT*, laryngeal elevation delay time; *CI*, confidence interval

## Discussion

The results showed that both PDT and LEDT were prolonged at mid-CRT and further extended at early post-CRT. Additionally, the timing of the swallowing response evaluated using PDT and LEDT was a significant factor for the swallowing safety with PAS score at mid-CRT and early postCRT. Many studies reported on the swallowing response of CRT in HNC, which showed that swallowing response or laryngeal sensation worsens months or years after CRT as a late side effect [7, 16]. Some patients in the extended period after RT also experienced a delayed swallowing response caused by fibrosis, or nerve injuries, which mediates the swallowing response, as late side effects [2, 17]. Conversely, other studies showed improvement of the swallowing response a few months after treatment compared with that before treatment [7, 9, 10]. This may be due to the delayed swallowing response caused by laryngeal sensation dysfunction due to tumors or pain in some HNC patients before treatment. By contrast, patients 1–3 months postCRT may experience less tumor-induced pain, late side effects, or acute side effects. Consequently, their swallowing response may exhibit superior functionality during this period. In contrast, acute side effects primarily affect the epidermis and mucosa. These effects are mainly due to cell depletion, inflammation, and hypoplasia, which can lead to mucositis and desquamation, accompanied by edema and erythema [18]. Lymphedema, which typically begins and progresses slowly a few weeks after the initiation of CRT, can affect both internal structures (pharyngeal and laryngeal mucosa) and external areas (skin and soft tissue). It may cause dysfunction in sensation and movement, leading to dysphagia [17, 19]. Thus, our findings suggest that during or shortly after CRT, a delayed swallowing response may occur, likely due to pharyngeal mucositis, lymphedema, and pain, rather than fibrosis or nerve injuries, which are more commonly problematic as late side effects. This delayed swallowing response likely contributes to the risk of aspiration and penetration as acute side effects. However, few studies focused on the swallowing response during and immediately after CRT, which is considered a critical period because patients are prone to aspiration due to acute side effects.

In a previous study with 916 dysphagia patients, PDT was a predictive factor for the development of aspiration pneumonia [20]. Appropriate timing for the initiation of pharyngeal swallowing, assessed using PDT or LEDT, is essential for both laryngeal closure and upper esophageal sphincter opening. Laryngeal closure is necessary to prevent premature bolus leakage into the laryngeal vestibule before the initiation of pharyngeal swallowing, and timely opening of the upper esophageal sphincter ensures the smooth transport of the bolus into the esophagus [21]. The delayed swallowing response is probably induced by reduced sensitivity and pain in the pharynx and larynx due to mucositis occurring during CRT, and the delayed swallowing response may contribute to aspiration or aspiration pneumonia. This finding may contribute to a better understanding of the physiological changes in swallowing function induced by CRT.

The subgroup analysis revealed possible influences of CRT on the swallowing response. First, as expected, PDT and LEDT were not affected in patients with nasal cavity or paranasal sinus cancer. By contrast, PDT and LEDT were prolonged in patients with other tumor sites in HNC, indicating that CRT on the pharynx, larynx, and neck likely influences the timing of the swallowing response. Second, the relationship between tumor size and the delayed swallowing response induced by CRT seemed tenuous, although advanced N stage appeared to be a risk factor for the delayed swallowing response. Bilateral neck irradiation is usually performed in patients with advanced N stage HNC, and the expansion of the irradiation field may affect the timing of the swallowing response. A previous study reported that 80% of participants experienced swallowing response when the bolus reached the level of the tongue base [22]. The glossopharyngeal nerve (cranial nerve IX) supplies the pharynx with general somatic afferent, general visceral efferent, special visceral afferent, and parasympathetic fibers [23]. Thus, oropharynx sensation which controlled by cranial nerve IX play important roles in the swallowing response. However, in our study, patients with oropharyngeal cancer or advanced T stage HNC did not show prolonged PDT and LEDT compared with other tumor sites, except the nasal cavity and paranasal sinus tumor, or T1–T3 patients. Although the oropharynx sensation play an important role in the swallowing response, it is a complex process involving the coordination of various muscles and nerves and is triggered by various stimuli, including sensory, tactile, olfactory, gustatory, and neurohormonal stimuli [23, 24]. Deterioration of the swallowing response may be more pronounced when CRT affects various areas, as opposed to localized irradiation.

Although T1–T3 patients had prolonged PDT and LEDT from mid-CRT to early post-CRT, T4 patients tended to have shortened PDT and LEDT. This indicates that bulky tumors, such as T4, obstructs the swallowing response before and during treatment, but a diminished or shrunken tumor may improve swallowing after CRT. In a previous study, delayed swallowing response observed in patients with advanced HNC improved after treatment [10]. In our study, T4 patients exhibited the longest PDT and LEDT at mid-CRT, indicating that they were affected by both the side effects of CRT and the tumor. Considering the swallowing function in patients with HNC, both the side effects of treatment and the tumor should be considered. Third, the absence of tube feeding experience during CRT was significantly associated with prolonged PDT and LEDT. Huggins et al. reported that feeding tubes affect the swallowing response in ten young, healthy adults, noting that the delayed swallowing response was more pronounced with wide-bore tubes compared to fine-bore tubes [25]. In contrast, another study found no significant difference in swallowing response between tube-in and tube-out conditions in stroke patients [26]. Thus, tube feeding, particularly through nasogastric tubes, can impact swallowing function, including the swallowing response, but the impact on swallowing safety appears minimal in most cases. In our study, tube feeding did not have a significant effect on swallowing response. In our study, tube feeding did not have significant effects on the swallowing response. This may be attributed to the use of fine-bore nasogastric tubes in our patients and/or the possibility that the impact of tube feeding was less significant than that of CRT.

Patients who used opioids at 40-Gy irradiation (mid-CRT) showed noticeable prolongation of PDT and LEDT from mid-CRT to early post-CRT. Multivariate linear regression analysis also showed that the opioid dosage was a significant factor in prolongation of PDT and LEDT from baseline. Patients using opioids most likely experience severe pain, indicating that they may have been affected by severe pharyngeal–laryngeal mucositis. Severe pharyngeal–laryngeal mucositis affects sensation in the pharynx and larynx, which affects the timing of the swallowing response. Moreover, these patients are susceptible to pharyngeal–laryngeal mucosa damage during CRT, which may continue to worsen after 40-Gy irradiation. Consequently, the swallowing response may have further deteriorated from mid-CRT to early post-CRT. This finding indicates that severe pharyngeal–laryngeal mucositis or opioid use could serve as predictive factors for subsequent swallowing dysfunction.

Our study has several limitations. First, because the frame rate of the VFSS was 7.5 frames/s, subtle changes in PDT and LEDT may not have been detected. Ideally, a frame rate of 30 frames/s should be used; however, we have identified clinically important changes even with 7.5 frames/s VFSS. Second, we evaluated the patient’s swallowing response with only a command swallow of 5 ml of liquid. Swallowing provocation test, VFSS with sequential swallowing, or chewing swallow could lead to other findings. Third, although we used multivariate analysis to identify factors affecting changes in PDT and LEDT, there may be unadjusted factors due to the retrospective design and limited sample size of this study. Further studies with larger sample sizes, higher frame rates (30 frames/s) for measuring PDT and LEDT, and comprehensive evaluation of variations in patients’ swallowing responses are essential.

In conclusion, our findings indicate that both PDT and LEDT were prolonged at mid-CRT and further prolonged after CRT. This deterioration of the swallowing response may contribute to swallowing safety, as evaluated by PAS. In addition, various irradiation and opioid use at 40-Gy irradiation may pose as risk factors for a deteriorated timing of the swallowing response. This study revealed insights into changes in swallowing physiology during and immediately after CRT. These findings can be applied to practical interventions, such as swallowing techniques and use of texture-modified diets, to prevent aspiration during and immediately after CRT. It may also provide implications for rehabilitation strategies aimed at enhancing swallowing function and ultimately improving patient outcomes.

## Data Availability

No datasets were generated or analysed during the current study.
